# Work-family conflict in times of crisis: The moderating roles of self-efficacy and time-based spousal support during pandemic-induced remote work

**DOI:** 10.1371/journal.pone.0348368

**Published:** 2026-05-05

**Authors:** Selda Coşkuner Aktaş

**Affiliations:** Department of Family and Consumer Sciences, Hacettepe University, Ankara, Türkiye; Public Library of Science, UNITED KINGDOM OF GREAT BRITAIN AND NORTHERN IRELAND

## Abstract

The COVID-19 pandemic accelerated the global transition to remote work, requiring working parents to rapidly adapt to home-based arrangements. As work and family boundaries became increasingly blurred, concerns regarding work–family conflict (WFC) intensified. Drawing on Conservation of Resources (COR) Theory, Social Cognitive Theory (SCT), and Asymmetric Boundary Permeability Theory, the present study examined the relationships among time-based spousal support, self-efficacy, work- and family-related time demands, and WFC. Data were collected online from 211 working parents in dual-earner families engaged in pandemic-induced remote work. An a priori power analysis confirmed that the sample size was sufficient to detect medium effects. Results indicate that work-to-family conflict was higher than family-to-work conflict. While work- and family-related time demands were not directly associated with conflict, time-based spousal support moderated the relationship between work-related time demand and work-to-family conflict. Self-efficacy did not moderate time-demand relationships but was directly associated with lower levels of both work-to-family and family-to-work conflict. These results highlight the significance of contextual and personal resources in shaping work–family dynamics under crisis-driven and externally imposed work arrangements.

## Introduction

Balancing work and family roles has been a longstanding focus in organizational and family research. Work–family conflict (WFC) arises when demands from work and family domains are incompatible, resulting in interrole conflict in which pressures from one domain interfere with participation in the other [1,2]. WFC is particularly salient for dual-earner families, in which both partners must manage professional responsibilities and household obligations simultaneously [3]. The challenge of meeting competing expectations in these domains may undermine well-being, damage relationships, and compromise performance across roles.

At the center of WFC research are the demands and resources associated with each domain. Demands refer to role-related obligations that necessitate sustained effort, such as intensive work schedules, tight deadlines, or childcare and household management responsibilities. Resources, by contrast, are assets that enable individuals to meet these demands successfully [4,5]. According to the Conservation of Resources (COR) theory [6,7], stress arises when valued resources are threatened, lost, or insufficient to meet rising demands. Resources may be contextual, such as support from close others, or personal, such as confidence in one’s ability to manage challenges. Within this framework, employment represents a critical condition resource in the work domain, while stable family functioning constitutes an essential condition resource in the family domain. WFC can therefore be understood as a stress process through which excessive demands in one domain undermine the resources indispensable for effective functioning in the other.

Demand–resource dynamics are particularly salient in remote work. Remote work, defined as performing job responsibilities outside traditional office settings, is frequently promoted as a strategy to enhance work–family integration by reducing commuting time and increasing flexibility [8]. When adopted voluntarily, remote work can offer greater autonomy and opportunities to manage boundaries. However, it may also introduce new demands, such as constant connectivity, blurred temporal boundaries, and intensified role overlap [9]. For dual-earner families, remote work presents both potential benefits and challenges, with outcomes dependent on the effective management of demands and mobilization of resources.

The COVID-19 pandemic fundamentally altered these dynamics by transforming remote work from a voluntary option into a mandatory and externally imposed arrangement. Unlike planned flexible work schedules, pandemic-induced remote work occurred abruptly, often in households unprepared for simultaneous caregiving, schooling, and professional responsibilities [10]. School closures, limited childcare services, and insufficient home infrastructure intensified both work- and family-related demands [11]. These challenges were particularly pronounced in Türkiye, where remote work practices had been relatively limited before the pandemic [12]. Turkish dual-earner families were therefore required to navigate heightened demands in a context of limited institutional support and evolving workplace expectations.

Despite extensive research on WFC, several gaps remain. Much of the telecommuting literature has focused on voluntary remote work in countries with established flexible work infrastructures [8,13]. Although pandemic-era studies have documented the rapid expansion of remote work, crisis-driven and externally imposed arrangements have not been systematically incorporated into traditional demand-resource frameworks [10,11]. Furthermore, research on WFC has examined contextual family resources, such as spousal support, and individual personal resources, such as self-efficacy [14–16], but these domains are often studied separately rather than within an integrated moderation framework, particularly in the context of crisis-induced remote work. This study addresses this gap by examining time-based spousal support, conceptualized as partner involvement in household and caregiving tasks, and self-efficacy as moderators of the relationship between work- and family-related time demands and WFC among dual-earner parents in Türkiye during the COVID-19-induced remote work period.

Grounded in COR theory’s [6,7] emphasis on resource mobilization under threat, the study also draws on Asymmetric Boundary Permeability Theory [17], which proposes that work pressures more readily intrude into family life due to their rigidity and external regulation, and on Social Cognitive Theory (SCT) [18], which conceptualizes self-efficacy as a central mechanism of personal agency and adaptive regulation. By integrating these perspectives, the study investigates (a) whether work-to-family or family-to-work conflict predominates under crisis-imposed remote work conditions, and (b) how contextual and personal resources are associated with the management of competing time demands across domains.

## Background and hypothesis development

### Remote work and work-family conflict

Remote work, defined as performing job responsibilities outside traditional office settings, has gained prominence with technological advances and the growing emphasis on work–family balance [8]. For dual-earner families, remote work has often been viewed as a valuable resource, as it may reduce commuting stress, increase autonomy, and support better alignment between work and family schedules [19]. At the same time, however, it may introduce new demands—such as ongoing connectivity, heightened expectations of availability, and blurred role boundaries—that intensify WFC [20,21]. These competing effects are especially marked for couples who simultaneously manage multiple professional, caregiving and household responsibilities.

These dual effects align with the logic of the Conservation of Resources (COR) Theory [6,7], which posits that individuals strive to obtain, retain, and protect valued resources—ranging from condition resources, such as employment and family stability, to personal resources like self-efficacy. For dual-earner families, employment continuity and a well-functioning household routine are critical conditions for resources. Stress emerges when these resources are threatened, lost, or require disproportionate investment to sustain. In this framework, remote work represents both promise and risk. It may free up resources such as time and autonomy, but it may also deplete them when competing demands escalate across domains.

Building on this, COR theory posits that individuals selectively mobilize and protect resources that are most central or most threatened in a given context. Under crisis-driven conditions such as pandemic-induced remote work, employment stability may become the primary resource to preserve. This conceptual framework helps explain why WFC may exhibit an asymmetric pattern under such circumstances. According to Pleck’s [17] Asymmetric Boundary Permeability Theory, work responsibilities tend to intrude into family life more readily than the reverse because they are less flexible, more urgent, and subject to stronger external pressures [22,23]. Collectively, these perspectives indicate that when employment is perceived as the most threatened resource, work-related pressures are more likely to intrude into family life than family pressures into work life. For dual-earner couples facing externally imposed remote work arrangements, this asymmetry may be especially pronounced, as inflexible job expectations constrain opportunities to reorganize household responsibilities.

The COVID-19 pandemic amplified these dynamics by transforming remote work from a voluntary flexibility arrangement into a mandatory shift for which workers were unprepared. Unlike negotiated telecommuting, pandemic-induced remote work was imposed suddenly, often in households lacking adequate infrastructure or childcare support [10]. School closures, expanded household and caregiving responsibilities, and blurred boundaries further intensified WFC [11,24], particularly for dual-earner couples simultaneously managing overlapping professional and household demands. Based on these theoretical and contextual considerations, the following hypothesis is proposed:

*H1: Work-to-family conflict is higher than family-to-work conflict under pandemic-induced remote work conditions.*

### Time demands, work-family conflict, and remote work

Role demands refer to the expectations and obligations that require sustained time and energy investment [4]. In dual-earner families, where both partners engage in paid employment while managing household responsibilities, time demands shape experiences across both work and family domains. In the work domain, long hours, tight deadlines, and expectations of constant availability have been associated with higher levels of work-to-family conflict [25]. Empirical research indicates that extended work hours are associated with greater work-to-family conflict, partly because they limit the time available for family engagement [1,26]. Similarly, family-related time demand—including caregiving and household responsibilities—may intrude on work obligations, causing family-to-work conflict [5,27]. In dual-earner households, where time must be negotiated across partners and roles, competing temporal pressures across domains may be associated with conflict in both directions.

From the perspective of Conservation of Resources Theory (COR) [6,7], time and energy investments represent finite resources. When disproportionate investment is required in one domain, individuals may experience resource depletion in the other domain, increasing their vulnerability to stress. In dual-earner households, where both employment continuity and family functioning represent valued condition resources, excessive time demands in either domain may threaten overall resource stability, particularly when clear boundaries between roles are lacking.

In traditional flexible remote work arrangements, temporal flexibility and reduced commuting are associated with lower levels of work–family conflict [28–31]. However, pandemic-induced remote work fundamentally altered this relationship. Instead of offering discretionary flexibility, externally imposed home-based work arrangements often intensified concurrent demands, with work and family responsibilities occurring within the same physical and temporal space [11,32]. Under these circumstances, the predictive value of time allocation for conflict may be diminished, suggesting that the mobilization of contextual and personal resources could play a more central role in shaping work–family dynamics. As a result, contextual and personal resources may influence both the strength and direction of the association between time demands and work–family conflict.

### Spousal support, work-family conflict, and remote work

Social support involves mobilizing resources within social networks to address functional needs during both routine and crisis situations [33]. In family contexts, spousal support constitutes a critical form of social support for managing work and family demands. Spousal support is typically categorized into emotional and instrumental components. Emotional support includes empathy, understanding, and encouragement that promote psychological well-being. In contrast, instrumental support consists of tangible assistance with childcare, housework, or daily tasks that directly reduce role-related demands [34,35]. Although both forms are essential, previous research has identified emotional and instrumental support as functionally distinct dimensions [33,36]. Emotional support facilitates psychological coping by providing empathy and reassurance, whereas instrumental support alleviates practical burdens by redistributing time and task-related effort across household roles.

This study adopts a narrower definition of spousal support, focusing specifically on time-based spousal support, defined as the extent to which a partner contributes time to childcare and household labor. This operationalization emphasizes observable behavioral involvement and tangible resource allocation rather than perceived emotional support. According to Conservation of Resources (COR) Theory [6,7], time-based contributions represent a contextual family resource that can buffer resource loss by preserving time during periods of heightened role strain.

This perspective is particularly relevant in the context of pandemic-induced remote work, where boundaries between work and family became blurred and external supports, such as childcare services or extended family assistance, were largely unavailable. Under these conditions, redistributing household labor within the household serves as a critical strategy for conserving valued resources, such as sustained employment and effective family functioning. Increased involvement by one partner in childcare and household responsibilities can preserve the other partner’s time and energy for work, thereby reducing work–family conflict (WFC).

Empirical research consistently demonstrates that increased spousal involvement in family responsibilities is associated with lower levels of WFC, higher marital satisfaction, and enhanced well-being [15,16,37]. Several recent studies conducted during the COVID-19 pandemic have operationalized spousal or partner support primarily through behavioral indicators, such as involvement in childcare and household labor, or through instrumental assistance related to time allocation, rather than perceived emotional support. For example, Chung et al. [38], Čikić and Rajačić [39], and Demir [40] examined partner support in terms of tangible contributions to household responsibilities within the context of remote work. Although terminology varied across these studies, their operational focus aligns conceptually with the current study’s definition of time-based spousal support as a behavioral and resource-based construct reflecting the redistribution of household labor.

Drawing on this literature and guided by the COR theory’s emphasis on resource mobilization under threat, this study examines whether time-based spousal support functions as a moderating resource, buffering the effects of work- and family-related time demands on work–family conflict. Accordingly, the following hypotheses are proposed:

*H2: Time-based spousal support moderates the relationship between work-related time demand and work-to-family conflict.*

*H3: Time-based spousal support moderates the relationship between family-related time demand and family-to-work conflict.*

### Self-efficacy, work-family conflict, and remote work

Self-efficacy, a central construct of Social Cognitive Theory (SCT) [18,41], refers to an individual’s belief in their capability to organize and execute actions required to manage specific demands. This construct functions as a self-regulatory mechanism that shapes perceptions of stressors, emotional regulation, and persistence in the face of challenges. Individuals with higher self-efficacy are more likely to perceive demanding situations as controllable, thereby enhancing adaptive coping and performance [18,41].

According to Conservation of Resources (COR) Theory [6,7], self-efficacy serves as a personal resource that individuals use to prevent resource loss and maintain balance across competing life roles. Whereas contextual resources such as spousal support mitigate external strain, self-efficacy enables individuals to mobilize effort, set goals, and sustain control over both work and family demands. Integrating SCT and COR clarifies how beliefs about personal capability inform resource management strategies that prevent loss spirals and foster resilience in high-demand contexts.

During the pandemic, the sudden convergence of work and family environments imposed significant pressure on working parents to fulfill professional, childcare, and household responsibilities concurrently. Under these conditions, self-efficacy becomes particularly important, as individuals confident in their ability to manage multiple roles are better equipped to regulate stress, conserve emotional resources, and reduce spillover conflict between work and family domains.

Empirical research consistently supports this perspective, demonstrating that higher self-efficacy is negatively associated with both work-to-family and family-to-work conflict [14,37,42–44]. Additionally, self-efficacy may serve as a moderating personal resource, reducing the relationship between time demands and work–family conflict by enhancing boundary regulation and emotional control. Therefore, the following hypotheses are proposed:

*H4: Self-efficacy moderates the relationship between work-related time demand and work-to-family conflict.*

*H5: Self-efficacy moderates the relationship between family-related time demand and family-to-work conflict.*

## Method

### Research model

This study synthesizes three theoretical perspectives: Conservation of Resources (COR) Theory [6,7], Asymmetric Boundary Permeability Theory [17], and Social Cognitive Theory (SCT) [18], to explain work–family conflict (WFC) among dual-earner families engaged in pandemic-induced remote work. COR Theory serves as the primary framework, positing that stress occurs when valued resources are threatened, lost, or require disproportionate investment to maintain. In this context, the central resources are employment and family functioning. Employment is compromised by family-to-work conflict, while family functioning is strained by work-to-family conflict. The predominant direction of conflict reveals which resource is more susceptible during crisis conditions.

Asymmetric Boundary Permeability Theory accounts for the likely predominance of work-to-family conflict. Work obligations are generally less flexible and subject to external regulation, which facilitates their intrusion into family life [22,23]. This structural asymmetry was particularly pronounced for dual-earner couples during pandemic-induced remote work, as professional and family boundaries converged and both partners navigated simultaneous demands within a shared environment.

Demands are conceptualized as time-based pressures that originate from both work and family domains:

- Work-related time demand is modeled as predictor of work-to-family (W → F) conflict, reflecting time pressures that reduce the availability of resources for fulfilling family responsibilities.
- Family-related time demand is modeled as predictor of family-to-work (F → W) conflict, reflecting time pressures that restrict the attention and resources available for work responsibilities.

Two categories of resources are proposed as potential buffers of these associations:

- Time-based spousal support is defined as a contextual family resource, characterized by tangible involvement in childcare and household responsibilities, as measured by the time a partner dedicates to these activities.
- Self-efficacy is conceptualized as a personal resource, reflecting individuals’ perceived capability to manage conflict between work and family domains (W → F and F → W conflicts).

**Fig 1:**
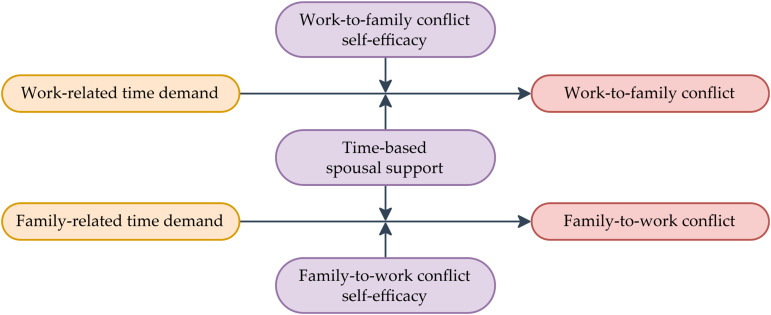
Conceptual model.

COR Theory elucidates the protective function of resources against stress, Asymmetric Boundary Permeability Theory highlights the structural imbalance between work and family domains, and SCT details the internal mechanisms by which self-efficacy operates as personal agency. Collectively, these perspectives clarify how structural asymmetry, contextual resources, and personal resources contribute to variations in WFC. The conceptual model (Fig 1) depicts the directional relationships among time demands, both directions of WFC, and the moderating effects of time-based spousal support and self-efficacy.

### Sample selection and data collection

This study examined work–family conflict (WFC) among working parents who transitioned to remote work during the COVID-19 pandemic. It was conducted as part of a larger mixed-methods project and adhered to the ethical principles of the Declaration of Helsinki. Ethical approval was obtained from the Hacettepe University Ethics Committee (E-90955707-200-00001417875, February 9, 2021).

Data were collected online from June 15 to July 30, 2021, using convenience sampling due to public health restrictions. Participants provided electronic informed consent before participation. Confidentiality and voluntary participation were emphasized, and participants were informed that results could be disseminated through open-access publication.

Eligibility criteria included being married, having at least one child, and having transitioned to remote work due to the pandemic. Single parents and married individuals without children were excluded. From the larger dataset, dual-earner households in which both spouses were employed were selected as the analytic subsample.

An a priori power analysis was conducted using G*Power 3.1.9.6 [45]. For the paired-samples t-test associated with H1, a minimum sample of 54 participants was required to detect a medium effect size (d = .50) with 95% power at α = .05. For the moderation analyses (H2–H5), a minimum of 119 participants was required; this higher threshold was used as the benchmark for adequacy.

Of 600 invitations distributed electronically, 231 responses were received (response rate = 38.5%). After excluding households in which one spouse was not employed, the final analytic sample consisted of 211 dual-earner families.

### Instrument

#### Work-to-family and family-to-work conflict scales.

Work-to-family (W → F) and family-to-work (F → W) conflict were measured using the 10-item scale developed by Netemeyer et al. [46], consisting of five items for W → F and five items for F → W. Participants rated each item on a 7-point Likert scale (1 = strongly disagree, 7 = strongly agree). Internal consistency coefficients reported by Netemeyer et al. [46] were.88 for W → F conflict and.86 for F → W conflict. The Turkish adaptation by Aycan and Eskin [35] reported internal consistency coefficients of.90 for W → F conflict and.89 for F → W conflict.

Sample items include: “The demands of my work interfere with my home and family life” (W → F conflict) and “The demands of my family or spouse/partner interfere with work-related activities” (F → W conflict). Higher scores indicate greater levels of conflict. Both W → F and F → W conflict were modeled as outcome variables in the moderated regression analyses.

#### Work-family conflict self-efficacy scale (WFC-SES).

Self-efficacy was measured using the Work-Family Conflict Self-Efficacy Scale (WFC-SES), developed by Cinamon (2003) and subsequently validated in its English-language version (Hennessy, K. D. [Unpublished]). The original instrument consists of two five-item subscales measuring confidence in managing work-to-family (W → F) and family-to-work (F → W) conflict. Items are rated on a 10-point scale ranging from 0 (complete lack of confidence) to 9 (complete confidence). In the original validation study, internal consistency coefficients were.83 (W → F) and.84 (F → W) [47]. The Turkish adaptation by Amanvermez and Denizli [47] retained the original two-factor structure with eight items (five W → F and three F → W) following item refinement. Internal consistency coefficients in the Turkish sample were.94 (W → F) and.93 (F → W) [47].

Sample items include: “How confident are you that you could fulfill your job responsibility without letting it interfere with your family responsibilities?” (W → F conflict self-efficacy) and “How confident are you that you could manage incidents in which family life interferes with work life?” (F → W conflict self-efficacy). Higher scores indicate greater confidence in managing work–family conflict. Self-efficacy was modeled as a personal moderating resource in the regression analyses.

#### Time-based spousal support.

Time-based spousal support was defined as tangible behavioral involvement in childcare and household labor. This operationalization captures observable time contributions rather than perceived emotional or relational dimensions of support. It was assessed with a single item: “On average, how many hours per week does your spouse allocate to childcare and housework?” Time-based spousal support was modeled as a contextual moderating resource for both directions of conflict.

#### Work-related and family-related time demands.

Work- and family-related time demands refer to the quantitative time commitments associated with work and family roles. Work-related time demand was measured as the average number of hours per day spent working, using a single item: “On average, how many hours per day do you allocate to your work?” Family-related time demand was measured as the average number of hours per week devoted to childcare and household tasks, using a single item: “On average, how many hours per week do you spend on childcare and household tasks?” Daily work hours captured the immediate temporal intensity of work engagement under remote conditions, whereas weekly family hours reflected cumulative caregiving and household involvement across the week. These time indicators were treated as objective demand variables in the moderation analyses.

### Data analysis

Data were analyzed using IBM SPSS Statistics (Version 23). Descriptive statistics (frequencies, percentages, means, and standard deviations) were computed for demographic and study variables. Hypothesis 1 was tested using a paired-samples t-test comparing mean levels of work-to-family (W → F) and family-to-work (F → W) conflict.

Hypotheses 2–5 were examined using moderated multiple regression analyses conducted with the PROCESS macro for SPSS [48]. Separate models were estimated for W → F and F → W conflict as the outcome variables. In the W → F conflict model, work-related time demand was entered as the predictor, with time-based spousal support and W → F conflict self-efficacy included as moderators. In the F → W conflict model, family-related time demand was entered as the predictor, with time-based spousal support and F → W conflict self-efficacy included as moderators.

All continuous predictors and moderators were mean-centered prior to computing interaction terms. Statistical inference for interaction effects was based on bootstrapped 95% confidence intervals using 5,000 resamples [48]. Sex, age of the youngest child, and organization type were included as control variables given prior evidence linking these factors to work–family conflict (WFC) under both conventional and pandemic-induced remote work conditions [49–56].

## Results

### Characteristics of participants

Of the 211 participants included in the analyses, 64.5% were female, and 35.5% were male. The mean age was 39.08 years (SD = 6.99). Regarding education, 58.3% held a university degree. The mean age of the youngest child was 6.45 (SD = 4.85). In terms of employment context, 64.9% worked in the public sector and 35.1% in the private sector (Table 1).

**Table 1:** Demographics of participants.

Variable	N	%	Mean	SD
**Sex**				
**Female**	136	64.5		
**Male**	75	35.5		
**Age**			39.08	6.99
**Education**				
**Up to high school**	17	8.0		
**Community college**	16	7.6		
**College**	123	58.3		
**Graduate**	55	26.1		
**Organization**				
**Public**	137	64.9		
**Private**	74	35.1		
**Age of the youngest child**			6.45	4.85
**Total**	**211**	**100.0**		

### Work-to-family and family-to-work conflict comparison

Table 2 presents descriptive statistics and the results of the paired-samples t-test comparing work-to-family (W → F) and family-to-work (F → W) conflict. W → F conflict was significantly higher than F → W conflict, t(210) = 7.09, p < .001. This finding indicates that, under pandemic-induced remote work conditions, work demands interfered with family life more than family demands interfered with work. Thus, H1 was supported.

**Table 2:** Means and paired-samples t-test results for work-to-family and family-to-work conflict.

Work-family conflict	Mean	SD	Mean difference	t	p
**W → F conflict**	21.13	8.92	3.72	7.091	<.001
**F → W conflict**	17.43	8.59			

### Preliminary analyses

Table 3 presents descriptive statistics, reliability coefficients (shown on the diagonal), and bivariate correlations among the study variables. Work-to-family (W → F) and family-to-work (F → W) conflict were positively correlated (r = .623, p < .01), indicating that higher conflict in one direction was associated with higher conflict in the other direction.

**Table 3:** Descriptive statistics and bivariate correlations.

Variables	1	2	3	4	5	6	7	8	9	10
**1. Sex**		−.403	−.048	−.112	.252**	−.270**	−.105	−.041	.067	.015
**2.Age of the youngest child**	−.403		.009	−.101	−.243**	−.152*	.057	.144*	.035	−.063
**3.Organization type**	−.048	.009		−.095	.059	.126	.113	.069	−.104	−.087
**4.Work-related time demand**	−.112	−.101	−.095		.113	.100	.020	.042	−.026	−.096
**5.Family-related time demand**	.252**	−.243**	.059	.113		.379**	.016	.000	−.070	.036
**6.Time-based spousal support**	−.270**	−.152*	.126	.100	.379**		.036	.034	−.068	−.011
**7.W → F conflict self-efficacy**	−.015	.057	.113	.020	.016	.036	(.92)	.782**	−.344**	−.326**
**8.F → W conflict self-efficacy**	−.041	.144*	.069	.042	.000	.034	.782**	(.90)	−.219**	−.394**
**9.W → F conflict**	.067	.035	−.104	−.026	−.070	−.068	−.344**	−.219**	(.94)	.623**
**10.F → W conflict**	.015	−.063	−.087	−.096	.036	−.011	−.326**	−.394**	.623**	(.92)
**Mean**		6.45		5.24	27.43	23.38	31.92	19.17	21.13	17.43
**SD**		4.85		3.09	21.08	24.02	8.07	5.15	8.92	8.59

Note. Sex was coded 0: women, 1: men, Organization type was coded 0: public, 1: private *p < .05, **p < .01.

W → F and F → W conflict self-efficacy were positively correlated (r = .782, p < .01) and were negatively associated with both W → F conflict (r = −.344 and r = −.219, respectively, p < .01) and F → W conflict (r = −.326 and r = −.394, respectively, p < .01). In contrast, time-based spousal support was not significantly correlated with either direction of conflict.

Family-related time demand was positively associated with time-based spousal support (r = .379, p < .01), suggesting that greater family time investment was linked to greater partner involvement in childcare and household tasks. Neither work-related nor family-related time demand was significantly associated with either conflict self-efficacy subscale. Additionally, work- and family-related time demands showed no significant direct associations with W → F or F → W conflict.

Overall, this pattern suggests that contextual and personal resources may operate through interaction effects rather than direct associations. These findings justify proceeding to the multiple moderation analyses.

### Multiple moderation analyses

The results of the multiple moderation analyses are presented in Table 4. The overall regression model predicting work-to-family (W → F) conflict was statistically significant, F(8, 202) = 4.51, p < .001, accounting for 15% of the variance. The interaction between work-related time demand and time-based spousal support was statistically significant (ΔR² = .019, p = .034), indicating that time-based spousal support moderated the association between work-related time demand and W → F conflict. Thus, H2 was supported. In contrast, the interaction between work-related time demand and W → F conflict self-efficacy was not statistically significant (ΔR² = .003, p = .384); therefore, H4 was not supported. Among the main effects, W → F conflict self-efficacy was significantly and negatively associated with W → F conflict (B = −.382, p < .001), whereas work-related time demand, time-based spousal support, and the covariates were not significantly associated with the outcome.

**Table 4:** Multiple moderation analyses predicting work-to-family and family-to-work conflict.

Outcome: W → F conflict	B	SE	t	p	ΔR²	95% CI	
						LL	UL
**Constant**	20.857	1.552	13.436	<.001		17.796	23.917
**Work-related time demand**	.304	.194	.178	.859		−.348	.417
**Time-based spousal support**	−.007	.026	−.272	.786		−.058	.044
**Int_1**	−.013	.006	−2.131	**.034**	**.019**	−.025	−.001
**W → F conflict self-efficacy**	−.382	.073	−5.257	<.001		−.526	−.239
**Int_2**	−.016	.018	−.872	**.384**	**.003**	−.052	.020
**Sex (covariate)**	.989	1.262	.783	.434		−1.500	3.478
**Age of the youngest child (covariate)**	.075	.122	.617	.538		−.166	.317
**Organization type (covariate)**	−1.152	1.234	−.934	.352		−3.585	1.281
**Outcome: F → W conflict**							
**Constant**	18.329	1.471	12.457	<.001		15.428	21.230
**Family-related time demand**	.023	.032	.717	.474		−.040	.085
**Time-based spousal support**	−.015	.029	−.504	.615		−.073	.043
**Int_1**	.001	.001	.797	**.426**	**.003**	−.001	.002
**F → W conflict self-efficacy**	−.665	.109	−6.102	<.001		−.879	−.450
**Int_2**	−.007	.006	−1.274	**.204**	**.007**	−.019	.004
**Sex (covariate)**	−.645	1.317	−.490	.625		−3.241	1.951
**Age of the youngest child (covariate)**	.021	.119	.179	.858		−.213	.256
**Organization type (covariate)**	−1.170	1.163	−1.006	.316		−3.463	1.124

Note. N = 211. CI = confidence interval; LL = lower limit; UL = upper limit. Int_1 represents the interaction between time demand and time-based spousal support; Int_2 represents the interaction between time demand and self-efficacy.

The overall regression model predicting family-to-work (F → W) conflict was also statistically significant, F(8, 202) = 5.20, p < .001, accounting for 17% of the variance. However, neither the interaction between family-related time demand and time-based spousal support (ΔR² = .003, p = .426) nor the interaction between family-related time demand and F → W conflict self-efficacy (ΔR² = .007, p = .204) was statistically significant. Accordingly, H3 and H5 were not supported. Consistent with the W → F model, F → W conflict self-efficacy was significantly and negatively associated with F → W conflict (B = −.665, p < .001), whereas family-related time demand, time-based spousal support, and the covariates were not significantly associated with the outcome (Table 4).

## Discussion

This study examined work–family conflict (WFC) among dual-earner families engaged in pandemic-induced remote work. Work-to-family conflict (W → F) was significantly higher than family-to-work conflict (F → W). Time-based spousal support moderated the relationship between work-related time demand and W → F conflict, but did not moderate F → W conflict. Self-efficacy did not moderate the relationship between time demands and either direction of conflict. However, higher self-efficacy was directly associated with lower levels of both W → F and F → W conflict. Furthermore, work- and family-related time demands were not directly associated with either direction of conflict. Collectively, these findings suggest that contextual and personal resources were more strongly associated with variations in WFC than objective time allocation alone.

The predominance of W → F conflict is consistent with Pleck’s [17] Asymmetric Boundary Permeability Theory, which proposes that work obligations are more likely to intrude into family life than the reverse because they are less flexible, more externally regulated, and subject to stronger institutional pressures [22,23]. For dual-earner couples working remotely, this asymmetry may have been particularly salient. Although work was physically carried out in the home, occupational expectations remained structured by supervisors, deadlines, and performance standards, while family tasks retained some internal flexibility. This structural imbalance offers a plausible explanation for the higher reported levels of W → F conflict and reinforces the idea that spatial proximity alone does not equal boundary symmetry.

The moderating role of time-based spousal support must be interpreted within this asymmetrical context. Greater partner involvement in childcare and household labor was associated with a weaker link between work-related time demand and W → F conflict, but not between family-related time demand and F → W conflict. From a Conservation of Resources (COR) perspective [6,7], this pattern is consistent with the proposition that individuals and families are likely to mobilize resources strategically to protect resources that are most central or most threatened. During pandemic-induced uncertainty, employment stability represented a critical condition resource. Accordingly, the redistribution of household labor may have been prioritized in ways that shielded work functioning from disruption. This interpretation corresponds to pandemic-era evidence suggesting that dual-earner households reorganized household labor to sustain work continuity under remote conditions [11,32]. Rather than demonstrating that spousal support reduces conflict in a causal sense, these findings indicate that time-based partner involvement is associated with differential levels of W → F conflict under heightened work salience.

This pattern is best understood within the broader cultural and institutional context of Türkiye. Although dual-earner families are increasingly prevalent, national time-use statistics demonstrate that women continue to perform the majority of unpaid household and caregiving labor, even among employed couples [57,58]. This persistent gender gap indicates that household responsibilities remain normatively feminized, despite increased female labor force participation. In this context, greater partner involvement in household labor during the pandemic likely represented a strategic adjustment to preserve employment functioning under uncertainty, rather than a substantive transformation of gender norms. Simultaneously, workplace practices in many Turkish organizations are characterized by expectations of constant availability, sustained performance, and responsiveness to supervisory demands, even in remote work settings. As institutional supports, such as in-person schooling, shifted to remote delivery and childcare facilities were temporarily suspended, families became increasingly reliant on internal resource coordination, while external organizational expectations remained largely inflexible. Under these circumstances, safeguarding work responsibilities, often regarded as financially and socially essential, may have taken precedence over minimizing F → W conflict. As a result, time-based spousal involvement may have been more closely associated with reduced W → F conflict than with changes in F → W conflict, where family role expectations likely remained stable and culturally embedded. This interpretation highlights the combined influence of resource mobilization processes (COR), workplace institutional pressures, and prevailing gendered role norms during crisis conditions.

Self-efficacy was significantly associated with both conflict directions, but did not moderate the effect of time demands. This finding aligns with Social Cognitive Theory (SCT) [18], which conceptualizes self-efficacy as a belief in one’s capability to exercise personal agency in managing challenging situations. From a COR perspective, self-efficacy functions as a personal resource that supports adaptive functioning under stress. Although COR theory often characterizes resources as buffers against stressors, resources may also exert direct protective effects by reducing overall vulnerability to strain. In this sense, higher self-efficacy is associated with more effective emotional regulation, boundary management, and role coordination, which, in turn, are linked to lower levels of WFC. These findings are consistent with research conducted during the pandemic, which demonstrated that work–family conflict self-efficacy is associated with reduced psychological distress and improved adjustment to remote work [42,59]. The absence of a moderating effect suggests that self-efficacy serves as a general resilience factor rather than altering the relationship between objective time demands and conflict.

Finally, the absence of direct associations between work- and family-related time demands and conflict warrants careful interpretation. Although prior research has frequently reported positive associations between time demands and WFC [1,26,28], the present findings may reflect the limitations of objective hour-based indicators in capturing subjective overload or perceived resource threat. In line with COR theory [6,7], stress reactions are activated primarily by perceived resource loss or threat rather than by quantitative time allocation alone. During pandemic-induced remote work, temporal and spatial boundaries were collapsed, and multiple demands were experienced simultaneously, potentially altering how individuals appraised and prioritized resource investment. Under such conditions, the impact of time demands appears to depend more strongly on the availability of contextual and personal resources than on raw time expenditure per se. Accordingly, the absence of main effects does not constitute theoretical disconfirmation but instead underscores the conditional nature of demand–conflict associations within crisis-driven remote work arrangements.

Taken together, the present findings illustrate how the three theoretical perspectives operate in a complementary and logically connected manner rather than as isolated explanations. COR theory provides the overarching framework by positioning work and family functioning as valued condition resources that are threatened under crisis conditions. Asymmetric Boundary Permeability Theory specifies the structural mechanism through which these resource threats become directionally imbalanced, explaining why W → F conflict predominates. SCT adds a personal-level dimension by identifying self-efficacy as a resource that shapes how individuals appraise and regulate competing demands. These perspectives converge on a shared resource-based logic. Structural boundary asymmetry shapes which domain is most vulnerable; contextual resources, such as time-based spousal support, influence how demands are redistributed within the household; and personal resources, such as self-efficacy, are associated with generalized resilience across both directions of conflict. The empirical pattern observed in this study—directional asymmetry, domain-specific moderation by spousal involvement, and a generalized association of self-efficacy with lower conflict—reflects this integrated, multi-level resource process.

## Conclusion

This study advances understanding of how working parents navigated work–family boundaries during pandemic-induced remote work. The findings indicate that variations in work–family conflict (WFC) under crisis conditions were more closely associated with the mobilization and integration of available resources than with time allocation. The greater prevalence of W → F conflict underscores the persistent dominance of work pressures, even when work is conducted at home.

This imbalance may reflect the prioritization of employment stability and external performance expectations over family needs during periods of uncertainty. Time-based spousal support emerged as a key contextual resource associated with reduced W → F conflict under work-related time demand, while self-efficacy functioned as an internal coping resource linked to lower levels of conflict in both directions. These findings indicate that maintaining balance in dual-earner households under crisis-induced remote work appears to depend more on the interplay between shared household involvement and personal agency than on temporal flexibility. This pattern underscores the necessity of considering structural, contextual, and personal resource processes concurrently when analyzing work–family dynamics under crisis-driven and externally imposed work arrangements.

## Theoretical and practical implications

This study adds to the work–family interface literature by integrating Conservation of Resources (COR) Theory, Social Cognitive Theory (SCT), and Asymmetric Boundary Permeability Theory to explain how working parents manage competing demands during crisis-driven remote work. Consistent with COR Theory [6,7], the findings indicate that contextual and personal resources—rather than time-based demands per se—were associated with variations in work–family conflict under high-demand conditions. Time-based spousal support, conceptualized as partner involvement in household labor, functioned as a contextual family resource associated with weaker W → F conflict, whereas self-efficacy operated as a personal resource associated with lower levels of conflict across both directions. In line with SCT [18], these findings highlight the role of self-regulated agency in sustaining adaptation when external supports are constrained.

The predominance of W → F conflict reinforces Asymmetric Boundary Permeability Theory [17], which posits that work pressures more readily intrude into family life due to their rigidity and external regulation. Even when conducted from home, work remained structured by organizational expectations, while family activities retained comparatively greater flexibility. This imbalance illustrates that spatial flexibility alone does not necessarily ensure boundary balance, particularly under involuntary or crisis conditions.

From a practical standpoint, the findings suggest the importance of both organizational and household-level interventions. Organizations may reduce W → F conflict by establishing clearer communication boundaries, setting realistic workload expectations, and supporting employees’ recovery time outside formal work hours. At the household level, promoting tangible forms of shared spousal involvement in childcare and household tasks can help buffer work pressures and protect family functioning. Furthermore, initiatives aimed at strengthening self-efficacy—such as mentoring, skills development, and stress-management training—may enhance employees’ capacity to regulate boundaries and sustain resilience in future crises or hybrid work arrangements.

## Limitations and future directions

This study has several limitations that provide directions for future research. First, its cross-sectional design prevents causal inferences and limits conclusions about the directionality and temporal stability of moderation effects. Longitudinal or diary-based studies could better capture how work–family conflict (WFC) fluctuates across evolving work arrangements and different stages of the family life cycle as demands and resources shift.

Second, the study utilized self-report measures collected from the same participants, which may introduce common-method bias and potentially inflate observed associations among variables. Future research could incorporate multi-source data (e.g., from partners or supervisors), objective workload indicators, and dyadic or couple-level designs that capture mutual resource mobilization processes. These approaches would provide a more comprehensive and less perceptually constrained understanding of work–family dynamics.

Third, the sample consisted of dual-earner parents in Türkiye, where remote work practices were relatively novel, gendered divisions of household labor persisted, and institutional supports were limited during the pandemic. Comparative studies across countries and policy environments could clarify how differing family policies, labor market structures, and cultural expectations influence the mobilization and effectiveness of work and family resources under varying structural conditions.

Fourth, time-based spousal support was measured using a single item reflecting time spent on household and caregiving tasks. Although this operationalization captured behavioral involvement, future research should employ multidimensional scales that distinguish between instrumental and emotional dimensions of support to examine their potentially distinct associations with both directions of conflict.

Finally, although self-efficacy showed a significant direct association with lower conflict, it did not moderate the relationship between time demands and WFC. Given the cross-sectional design, it remains unclear whether enhancing self-efficacy would causally reduce work–family conflict. Experimental and intervention-based research has demonstrated that self-efficacy can be strengthened through targeted mastery experiences [60], suggesting that future research could investigate whether similar approaches produce subsequent reductions in domain-specific conflict. Such designs would allow stronger causal inferences regarding the role of personal agency in resource mobilization processes under work–family strain. Future research could also examine how self-efficacy interacts with related personal resources—such as resilience, boundary management skills, or emotion regulation capacities—to better understand when and how personal agency influences domain-specific conflict under crisis conditions.

## Supporting information

S1 File De-identified dataset. (XLS)

S2 File Data description note. (DOCX)
