# Supplementary material for: Work-family conflict in times of crisis: The moderating roles of self-efficacy and time-based spousal support during pandemic-induced remote work
Source: PLoS One. 2026 May 5;21(5):e0348368. doi: 10.1371/journal.pone.0348368 (PMC13143121; doi:10.1371/journal.pone.0348368)
Supplement: S2 File — (DOCX) [file pone.0348368.s002.docx]

**Data Description Note**

Supporting Information file S1_Data contains the minimal de-identified dataset necessary to replicate all analyses reported in the manuscript. Variable names correspond to those described in the Methods section. Composite variables were computed as the mean of their respective items.
